# Left Ventricular Noncompaction in Children: The Role of Genetics, Morphology, and Function for Outcome

**DOI:** 10.3390/jcdd9070206

**Published:** 2022-06-30

**Authors:** Sabine Klaassen, Jirko Kühnisch, Alina Schultze-Berndt, Franziska Seidel

**Affiliations:** 1 Max Delbrück Center for Molecular Medicine in the Helmholtz Association (MDC), 13125 Berlin, Germany; jirko.kuehnisch@mdc-berlin.de (J.K.); alina.schultze-berndt@charite.de (A.S.-B.); seidel@dhzb.de (F.S.); 2 Experimental and Clinical Research Center, a Cooperation between the Max Delbrück Center for Molecular Medicine in the Helmholtz Association and Charité Universitätsmedizin Berlin, 13125 Berlin, Germany; 3 DZHK (German Centre for Cardiovascular Research), Partner Site Berlin, 10785 Berlin, Germany; 4 Department of Paediatric Cardiology, Charité Universitätsmedizin Berlin, Corporate Member of Freie Universität Berlin and Humboldt-Universität zu Berlin, 13353 Berlin, Germany; 5 Department of Congenital Heart Disease-Paediatric Cardiology, German Heart Institute Berlin, 13353 Berlin, Germany

**Keywords:** noncompaction, cardiomyopathy, congenital heart disease, genetics, children

## Abstract

Left ventricular noncompaction (LVNC) is a ventricular wall anomaly morphologically characterized by numerous, excessively prominent trabeculations and deep intertrabecular recesses. Accumulating data now suggest that LVNC is a distinct phenotype but must not constitute a pathological phenotype. Some individuals fulfill the morphologic criteria of LVNC and are without clinical manifestations. Most importantly, morphologic criteria for LVNC are insufficient to diagnose patients with an associated cardiomyopathy (CMP). Genetic testing has become relevant to establish a diagnosis associated with CMP, congenital heart disease, neuromuscular disease, inborn error of metabolism, or syndromic disorder. Genetic factors play a more decisive role in children than in adults and severe courses of LVNC tend to occur in childhood. We reviewed the current literature and highlight the difficulties in establishing the correct diagnosis for children with LVNC. Novel insights show that the interplay of genetics, morphology, and function determine the outcome in pediatric LVNC.

## 1. Introduction

Cardiomyopathies (CMP) are myocardial diseases of which dilated (DCM) and hypertrophic (HCM) CMP are the most common subtypes. Restrictive (RCM), left ventricular noncompaction (LVNC), and mixed cardiomyopathies occur less frequently. Arrhythmogenic right ventricular cardiomyopathy (ARVC) is a rare disorder [1]. According to the American Heart Association (AHA), CMP are associated with mechanical and/or electrical dysfunction, and are classified into primary (genetic, nongenetic, acquired) and secondary forms [2]. The European Society of Cardiology groups CMP into the subtypes DCM, HCM, RCM, ARVC, and unclassified CMP, the latter includes LVNC. Further subclassification into familial/genetic and non-familial/non-genetic forms are part of the ESC classification [3]. This illustrates that classification of the LVNC phenotype is highly controversial and no general agreement exists. Examples of monogenic inheritance of the phenotype are frequent, but the association with CMP and other phenotypes rather suggest that the genetic architecture of LVNC is far more complex. Genetic background might be important for the development of noncompacted as well as compacted myocardium [4]. More recent data now point to isolated LVNC as a distinct phenotype which does not need to be pathologic [5]. LVNC has been shown to have a genetic cause in DCM, HCM, RCM, ARVC, the channelopathies, and congenital heart defects (CHD) [6,7,8]. More specifically in the pediatric population, LVNC has been identified with systemic entities such as Barth syndrome, chromosome 1p36 deletion syndrome, and CMP resulting from neuromuscular, metabolic, or mitochondrial disease [4,9,10]. We reviewed the literature to help identify the genetic and clinical features to determine outcome of LVNC in children.

## 2. Phenotype and Definition

There are many difficulties with the definition of the phenotype of LVNC. It is beyond the scope and not the intention of this review to define the history of several decades of the different definitions the phenotype of LVNC and we refer to several excellent previous reviews [4,8,11]. The most widely used definition of LVNC is still the one by Jenni et al. from 2001 [12]. The particular morphology, which was detected by echocardiography, mostly in adult patients with end-stage heart failure, with the technological advances in echocardiography raised much attention since then. As Jenni et al. described it, LVNC is a ventricular wall anomaly morphologically characterized by numerous, excessively prominent ventricular trabeculations and associated are deep intertrabecular recesses that are continuous with the left ventricular (LV) cavity [12]. A two-layer structure is present, with a compacted thin epicardial layer and a much thicker non-compacted endocardial layer. A maximal end systolic ratio of non-compacted to compacted layer of >2 is diagnostic (NC/C > 2) [12]. The extent of compacted to noncompacted myocardium does not lead to clinical manifestations and CMP *per se*. Regional ventricular hypokinesia was not restricted to the non-compacted segments in the original description [12]. Coexisting cardiac abnormalities are absent in *isolated* LVNC [12]. Of note, “*isolated*” in the original definition by Jenni et al. did not exclude the diagnosis of CMP, but was referring to the absence of CHD. Cardiac magnetic resonance (CMR) imaging was used in the following years to describe ratios between thickness, mass, or volume of NC/C [13,14]. The role of the thin compacted layer, which is used for the definition of LVNC, was not specifically defined. The compacted layer can be normal but also be disproportionately thinner than in controls [15]. Normal or abnormal thickness of the compact myocardium cannot be taken as a morphologic marker for LVNC. Rather, NC/C values (the ratio) need to reach cut-off values for the diagnosis [4,12]. Different other methods and normal reference values are available due to the lack of consensus criteria for LVNC [4,11]. The term “hypertrabeculation/noncompaction” is disputed due to interobserver disagreement [16]. Right ventricular noncompaction is a difficult morphology because an increased trabeculation is physiological in the right ventricle [11]. As for now, there are no morphologic markers or features that could predict the clinical impact of LVNC, whether it is a CMP or not. 

## 3. Pathogenesis and Genetics

Many different terms and abbreviations used for noncompaction CMP and the noncompacted morphology are used and relate to the possible pathogenesis [11]. In 1990, Chin et al. suggested the term “isolated noncompaction of the left ventricular myocardium (INVM)”. It was assumed that the myocardial anomaly occurred as a result of an arrest of the normal compaction process during embryonal endomyocardial morphogenesis [17]. 

### 3.1. Developmental Aspects 

Two developmental processes were described that govern the development of the normal myocardium. The first event is ventricular trabeculation which begins around week 4 of human development, the second is myocardial compaction during weeks 5 to 8 [18]. As demonstrated by several investigators, the Notch signaling pathway is critical for these developmental processes [19]. Most recently, two more unique genetic pathways were found in LVNC by genetic pathway analysis in humans: cardiomyocyte differentiation via BMP receptors and factors promoting cardiogenesis in vertebrates [20]. Quite the contrary was postulated by others, namely that LVNC may not be the result of an arrest in the compaction process at all. Instead LVNC could be the result from the compacted myocardium of the ventricular wall that grows into the ventricular lumen forming the trabeculae [21]. Jensen et al. found that left ventricular excessive trabeculations (LVET) are not of embryonic origin [21]. A reduction of the trabecular layer was never documented in humans [22]. Instead, they propose that the trabecular layer grows slower than the compact layer in later human development and this modulates wall morphology [22]. As there is no clear evidence for compaction on the developmental level the term “noncompaction” might need to be replaced [5]. Higher spatial resolution may clarify the prevalence and clinical significance of “LVET” [23]. 

What is the function of trabeculae in the adult heart, if we understand that excessive trabeculations might be detrimental? There is actually evidence that trabeculae are determinants of cardiac performance in adult hearts. The physiological function of trabeculae in the human heart was studied, using the UK Biobank, MRI data with fractal analysis, and modeling. The relationship of trabeculae-associated genomic loci with the risk of cardiovascular disorders was studied [24]. Reduced trabecular complexity was causally associated with the risk of heart failure. Trabeculae were important for cardiac contractility in both healthy and failing hearts.

Considerable evidence now favors the assignment of a mere phenotype to LVNC and genetic investigations are necessary to assign the possible pathogenicity of the phenotype [25,26]. 

### 3.2. Molecular Genetics

How could molecular genetic analysis help to determine the clinical outcome of the individuals affected with LVNC? The first genetic abnormality to cause LVNC without evidence of CHD was mutation in the X-linked gene *TAFAZZIN* [10]. Linkage analysis identified several autosomal dominant LVNC loci in families with LVNC phenotype, composed of individuals with and without signs of CMP [8,27]. Candidate gene analysis revealed that LVNC was associated with rare Mendelian variants in sarcomere genes [7,28]. In the majority of cases, LVNC is inherited in an autosomal dominant mode with heterozygous pathogenic variants leading to pediatric or adult LVNC [7]. Autosomal recessive inheritance or compound heterozygosity with early, severe disease phenotype were noted as well [29,30,31]. Compared with adults, children more frequently have X-linked disease, a mitochondrial inherited defect or chromosomal anomalies than adults [32]. 

In addition to genes coding for sarcomeric proteins, there are those coding for components of the nuclear envelope and Z-band, ion channels, sarcolemma proteins, and genes involved in signaling pathways or gene transcription. These genes associated with LVNC overlap with the genes causing CMP, therefore genetics suggest that LVNC-CMP may also be called HCM with LVNC or DCM with LVNC [33].

From the sarcomere genes, *MYH7* was reported first and most frequently in adults and in children with LVNC. *ACTC1*, *TTNT2*, and *TPM1* were also described as LVNC genes [7,34]. Later sequencing of *TTN* became feasible [35]. In individuals with HCM and LVNC, mutation of the gene *MYBPC3* was frequent [28,33,34]. The incidence of MACE is overall comparable between genotype-positive and genotype-negative LVNC individuals [34,36]. The mechanisms by which defective sarcomere genes cause LVNC remain to be elucidated [4]. In a systematic review in adult LVNC, genotype-phenotype correlations were assessed [32]. Single missense mutations in sarcomere genes were the main causes for the phenotype, and 48% of the of the sarcomere mutations were in *MYH7* [32]. *MYH7* and *ACTC1* mutations had a lower risk for MACE than *MYBPC3* and *TTN* [32].

The spectrum of genetic variants identified in LVNC patients is heterogeneous because of the differences in enrolment criteria and site of investigation. The description of LVNC was first derived from a cohort of patients from tertiary centers with an obvious CMP. This would explain the relatively high yield in genetic testing in this preselected population [35,37]. The differences in genetic yield are caused by the current problems with the diagnosis of LVNC with clinical manifestations that range from asymptomatic CMP to heart failure with major adverse cardiac events (MACE). With next generation sequencing (NGS) a genetic cause is identified in overall up to 30% of adult index cases, but with up to 44% even more frequently found in children [32,38,39]. 

Especially in children, LVNC is often first detected as part of the clinical investigation into an unclear genetic syndrome, muscular dystrophy, or mitochondrial myopathy [4]. Genetic syndromes with LVNC have been identified in patients with chromosomal anomalies, aneuploidies, and microdeletion syndromes, and most represent isolated sporadic cases. Chromosome 1p36 deletion syndrome is the most common human terminal deletion syndrome and LVNC is identified in 23% of individuals. The CMP in 1p36 deletion syndrome and in a proportion of non-syndromic LVNC and DCM is caused by *PRDM16* deletion or mutation, respectively [9]. 

In fact, to date more than hundred genes were described to be associated with LVNC [4,20]. From all the available literature on possible genetic associations with LVNC the study by Mazzarotto et al [40]. should be explained in more detail. It was determined whether or not LVNC was a distinct disease or a phenotype of CMP by using rare variant burden analysis. LVNC was characterized by a large overlap with DCM/HCM, but was also associated with distinct noncompaction and arrhythmia etiologies. LVNC-specific variant classes were truncating variants in *MYH7*, *ACTN2*, and *PRDM16*). Non-truncating (transmembrane) variants in *HCN4* and structural variants in *RYR2*, both variant classes in genes involved in arrhythmia phenotypes were specific for LVNC. LVNC-specific variants probably explain 5–10% of cases. Significant gene-disease associations were detected, but no significant excess of rare variation was observed for the majority of the analyzed genes. These findings were very similar to previous findings for HCM and DCM [40,41]. Interestingly, a significant excess of rare variants in LVNC cases compared with gnomAD (https://gnomad.broadinstitute.org/; accessed on 18 April 2022) was observed for the variants in many of the sarcomere genes first reported to be associated with LVNC [7,28,34]. *MYH7* (TV) with an anticipated mechanism of haploinsufficiency have generally been considered to be non-pathogenic in DCM or HCM, but was proposed as a pathomechanism in LVNC. The most frequent *MYH7* (TV) variant in LVNC is the splice site variant c.732+1G>A which does not seem to be a founder variant [7,40]. It is important to mention that in ClinGen (https://clinicalgenome.org/; accessed on 18 April 2022) LVNC is not curated as a specific disease entity and it awaits further consideration if or how these specific LVNC variant classes will be assigned.

### 3.3. Functional Models

Several animal models were created to discover the underlying mechanisms of LVNC [8]. Unfortunately, only in a few genes investigated by these genetic models, pathogenic genetic variants were found in the corresponding human genes. The pathology of LVNC is difficult to recapitulate in murine models [42]. Genes which showed a LVNC phenotype in humans and demonstrated diverse cardiac phenotypes in murine models were *PRDM16, MIB1, TBX20*, *NKX2-5,* and *TAFAZZIN*. One of the CMP genes found by Mazzarotto et al. to be causative for LVNC only, and curated with limited evidence in ClinGen for DCM (https://clinicalgenome.org/; accessed on 18 April 2022) has recently been investigated in more detail, namely *PRDM16*. Both, contractile dysfunction and partial uncoupling of cardiomyocytes were the result of modelling of *PRDM16* haploinsufficiency and a human truncation mutant in zebrafish [9]. There was also evidence of impaired cardiomyocyte proliferative capacity [9]. Genome editing of *PRDM16* in human iPSC-derived cardiomyocytes (iPSC-CMs) recapitulated the proliferative defects associated with LVNC at the single-cell level [43]. Varying expression levels of mutant allele-specific mRNA expression between mild DCM and LVNC were used to explain the spectrum of clinical manifestations in LVNC [43]. Two murine genetic models with cardiomyocyte-specific Prdm16-null mutation were described [44,45]. In one model, using the Myh6-Cre driver, cardiac conduction abnormalities and a CMP phenotype were found [45]. Another mouse model deactivating Prdm16 with the Mesp1-Cre driver demonstrated predisposition to heart failure in response to metabolic stress [44]. Genetic lineage tracing revealed that compact myocardial myocytes are more proliferative than trabecular cardiomyocytes [46]. Impaired proliferation and expansion of the compact myocardium with premature cardiomyocyte maturation was suggested in the developing mouse heart to underlie LVNC [47]. In a more recent study, two new conditional *Prdm16* knock out models recapitulated the LVNC phenotype with LV-specific dilatation and dysfunction [48]. In summary, the correct timing and spatial organization of cardiomyocyte proliferation and maturation is indispensable for normal left ventricular wall morphology and function.

Murine models were also used to test the hypothesis that LVNC may result from a combination of genetic variants. Evidence for oligogenic inheritance of LVNC was shown for a combination of three inherited heterozygous missense variants in the MKL/myocardin-like protein 2 (*Mkl2*), *Myh7*, and the transcription factor NK2 homeobox 5 (*Nkx2-5*) underlying familial LVNC. These triple compound heterozygous mice recapitulate signs of human LVNC [49]. Oligogenic inheritance was also supported by a study in which an increasing number of genetic variants in patients with LVNC strongly correlated with several markers of disease severity [50].

## 4. Diagnosis

The diagnosis of LVNC is made according to the morphologic criteria and made irrespective of the presence of LV systolic dysfunction or dilation [4,12]. This means that LVNC is a distinct but not necessarily pathological phenotype [51,52]. A wide spectrum of individuals meet the diagnostic criteria of LVNC without an underlying CMP. The genetic causes of LVNC are heterogeneous, reflect the underlying CMP such as DCM or HCM and most of them are not specific for the diagnosis of LVNC. Some acquired and reversible forms of LVNC strengthen that LVNC may be a physiologic, reversible phenotype [53,54,55]. LV trabeculations occur in response to increased LV loading conditions in pregnancy [56]. In one study, a high proportion of young athletes fulfil criteria for LVNC which suggests that the current diagnostic criteria, if used in an elite athletic population, are non-specific [57]. Ethnic considerations were described in which LVNC was more common in black individuals among an adult population fulfilling LVNC criteria supporting that LVNC might be a physiologic phenotype [26,58]. 

In an attempt to separate the individuals with pathologic from physiologic phenotype the term LVNC-CMP was introduced to specify those LVNC individuals with CMP [26]. When LV dilation and dysfunction are present, which are defining DCM, the diagnosis of LVNC-CMP may be more supported by using the DCM phenotype than by the trabecular morphology. In those cases, the diagnosis of DCM with LVNC morphology might be more suitable and practical. Both possibilities, either LVNC or CMP as the leading descriptor, provide the phenotype and diagnosis [4]. The morphology should be clearly described and practical to understand. 

### 4.1. How to Reach and Confirm a Diagnosis

**Figure 1:**
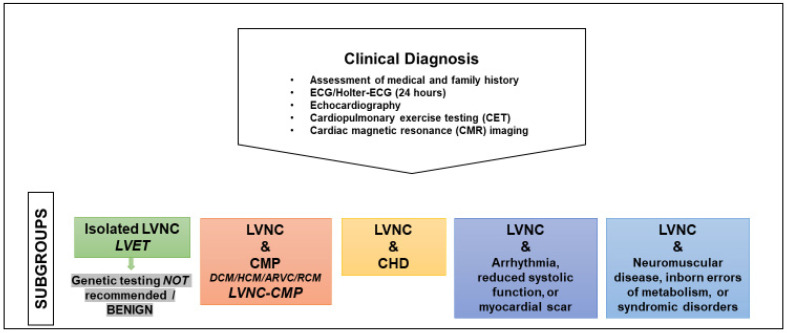
Clinical diagnosis and subgroups in pediatric LVNC. ARVC, arrhythmogenic right ventricular cardiomyopathy; CHD, congenital heart defects; CMP, cardiomyopathy; DCM, dilated cardiomyopathy; HCM, hypertrophic cardiomyopathy; ECG, electrocardiogram; LVET, left ventricular excessive trabeculation; LVNC, left ventricular noncompaction; LVNC-CMP, left ventricular noncompaction cardiomyopathy; RCM, restrictive cardiomyopathy.

The different subtypes of pediatric LVNC are summarized in Figure 1. For the diagnosis of a CMP in the presence of LVNC, the following investigations are usually performed [26,36,38,59].

- Assessment of medical and family history
- Electrocardiography (ECG)
- Echocardiography with functional assessment of LV size and function
- Holter-ECG (24 h)
- Cardiopulmonary exercise testing (CET)
- Cardiac magnetic resonance (CMR) imaging
- Genetic testing

Family history should include a three-generation pedigree. ECG changes are usually unspecific and arrhythmias detected by Holter-ECG may include ventricular ectopic beats and sustained or non-sustained ventricular tachycardia. Echocardiography data show LV dilatation and/or systolic/diastolic dysfunction. An early marker of systolic impairment is a reduced global longitudinal strain (GLS). CET is a measure for cardiovascular performance and possible arrhythmias. CMR imaging is used to characterize LV size and function and markers such as late gadolinium enhancement (LGE) are able to provide noninvasive tissue characterization and measure of fibrosis. Genetic testing should only be performed, if a CMP is diagnosed clinically.

With respect to subclinical CMP which can be detected by the newer imaging technologies there is still some uncertainty and no general agreement but the following is suggested. If strain is abnormal with normal LV-EF, the criteria for a CMP are not fulfilled according to the current AHA/ESC guidelines. These patients do not have CMP. LVNC with normal LVEF and without any other cardiovascular abnormalities, isolated LVNC, should be named LVET (Figure 1). Subclinical CMP (abnormal strain, clinically asymptomatic) requires clinical monitoring with echocardiography but genetic testing is not recommended.

### 4.2. The Importance of Imaging Modalities

**Figure 2:**
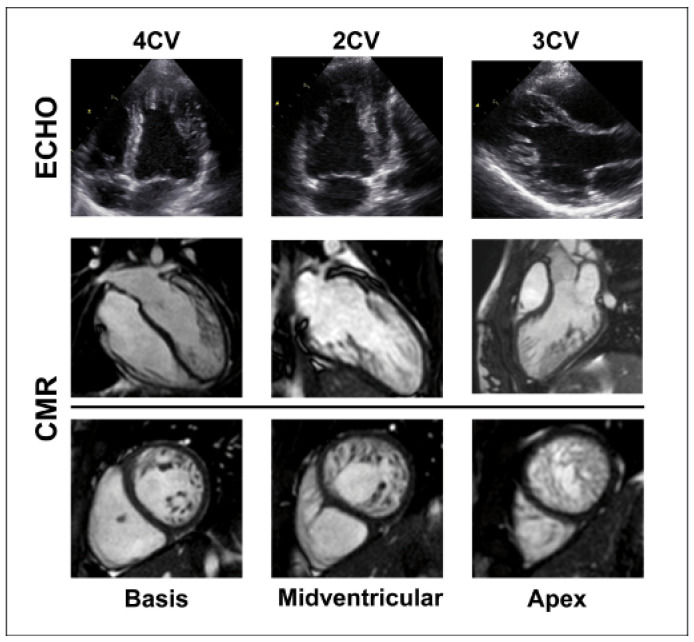
Echocardiography and cardiovascular magnetic resonance (CMR) imaging in left ventricular noncompaction (LVNC) of a 14-year-old girl with LVNC. The upper and middle panel show 4, 2, and 3 chamber views (CV) of representative echocardiographic and CMR images, respectively. The lower panel depicts the CMR short axis view at the basis, midventricular site, and apex of the same heart.

Both imaging modalities, echocardiography and CMR imaging, are essential to determine the morphology (Figure 2). The echocardiographic criteria by Jenni et al [12]. remain the gold standard for the LV phenotype “noncompaction” and are most widely used [11]. 

Several studies have proposed CMR criteria to improve the diagnostic sensitivity and specificity [11]. Most of these studies have integrated Jenni’s criteria. Petersen et al. were the first to propose CMR criteria: “an NC/C ratio greater than 2.3 in a long axis end-diastolic image, in at least two consecutive segments” [14]. Further CMR studies demonstrated that the numerical cutoff may not be sufficient for the diagnosis and other biomarkers or deeper phenotyping was necessary [60]. Nevertheless, CMR provides important long-term prognostic information. CMR may differentiate patients with high rates of cardiac events from those with an excellent prognosis [54]. 

LV dilatation, systolic dysfunction, and late gadolinium enhancement (LGE) were independent predictors of poor outcome [54]. Interestingly, the extent of LV trabeculation failed to have a significant prognostic impact in this study [54]. In another study, at multivariate analysis LGE was the only independent predictor of impaired systolic LV function [61]. LGE was identified as an additional risk factor in adult LVNC patients without severe LV systolic dysfunction [36]. To guide the management of patients, a risk prediction model was developed and validated [36]. 

Myocardial extracellular volume (ECV) reflects diffuse myocardial fibrosis with CMR T1 mapping. Zhou et al. found elevated native T1 values in LVNC patients compared to normal controls [62]. LGE negative patients had elevated native T1 compared to normal controls which suggested that native T1 mapping can be applied to detect myocardial fibrosis in LVNC patients earlier than by LGE imaging [62]. In comparison, ECV was normal in most of the pediatric LVNC patients and was not related to cardiac function [63]. This was explained by their younger age and that the patients were clinically asymptomatic [63]. Midwall fibrosis (MWF) can be detected by LGE CMR and predicts adverse outcome in adults with non-ischemic DCM [64]. MWF was common in pediatric DCM, but was unable to predict heart failure in the pediatric study [65]. Data on MWF in LVNC would be of special interest and are not available so far.

Speckle-tracking echocardiography (STE) can detect subclinical changes in LV function, and may complement the echocardiographic assessment of LVNC patients in clinical routine. Although altered LV deformation in LVNC phenotype by STE does not enable the diagnosis of a definite CMP it can be helpful in clinical assessment. Particularly circumferential parameters are impaired in LVNC and can help to predict cardiovascular outcomes of LVNC [66]. 

LV trabeculation was found to be correlated with reduced myocardial deformation indexes, even if left ventricular ejection fraction (LV-EF) was normal [67]. Children with LVNC and normal LV function myocardial deformation patterns measured by tissue tracking analysis are significantly impaired compared to controls [68,69]. Rigid body rotation (RBR) is characterized by impaired LV twist with apex and base rotating in the same direction and can be used as a measure of cardiac mechanics in echocardiography and CMR. RBR was associated heart failure and MACE in pediatric DCM [65]. In adult LVNC patients, increased RBR was shown in LVNC with preserved EF when compared with controls [70]. In children, LV twist had good predictive value in diagnosing LVNC in young patients [71]. In summary, the findings indicate that ECV, RBR, and tissue tracking analysis may serve as novel markers of disease severity and permit earlier detection of LV functional impairment in pediatric LVNC.

### 4.3. Indication for Genetic Testing

With regard to genetic testing the American Heart Association, the Heart Failure Society of America, and the American College of Medical Genetics and Genomics propose guidelines following a common line [25,72,73]: “Genetic testing should be considered in individuals with CMP co-occurring with LVNC according to the recommendations for the associated CMP [73]. Genetic testing is not recommended when the LVNC phenotype is identified in asymptomatic individuals with otherwise normal cardiovascular phenotype (Figure 1) [25,72]. The same causes of familial HCM and DCM common in adults are also found throughout childhood and may be associated with LVNC [25]. Mitochondrial disease genes, metabolic causes of CMP, and genetic syndromes should be tested following the suspected underlying disorder according to the cardiovascular phenotype and additional extracardiac clinical features of the individual” [25].

## 5. Prognosis and Outcome

Because existing diagnostic criteria lack specificity prognosis and outcome are difficult to determine, LVNC includes both, physiological conditions or adjustments and pathological disease [74]. In cohorts retrieved from tertiary centers, it has repeatedly been demonstrated that LVNC was associated with an increased risk of heart failure, systemic embolisms, and ventricular arrhythmias, which are the clinical symptoms and events that guide therapy [38,75]. Reduced LV ejection fraction was the variable most strongly associated with MACE in adult and pediatric patients with LVNC [36,39,76,77].

### 5.1. Adult and Paediatric LVNC Outcome

Data on outcome of pediatric LVNC are scarce and therefore studies of adult patients are often taken into consideration. Overall survival was reduced in patients with LVNC compared with the expected survival of age- and sex-matched US population [76]. In this study by Vaidya et al. however, survival rate in the individuals with normal LV-EF and isolated apical noncompaction was comparable with that of the general population [76]. In multivariate cox regression analysis in adult LVNC, age, LV-EF < 50%, and extent of LVNC were significantly associated with all-cause mortality [76]. In a metanalysis Aung et al. systematically searched 28 eligible studies enrolling 2501 adult LVNC patients, mostly all studies with patients with reduced LV-EF, and reported the adverse outcomes [77]. Patients with LVNC had a similar outcome when compared with DCM patients [77]. LV-EF, not the extent of trabeculation, was a factor of adverse outcomes in LVNC patients [77]. In another large adult LVNC study population, the risk of MACE was related to factors such as familial aggregation, male sex, cardiovascular risk factors, older age at diagnosis, certain ECG abnormalities, and LGE on CMR, and like in so many other studies, to reduced LV-EF [36]. From all these studies, no concrete conclusions can be made for pediatric LVNC.

Genetic factors play a more important role in children than in adults with LVNC and severe forms of LVNC tend to be diagnosed in childhood [8,38]. LVNC had a high mortality rate in children in a tertiary center, resulting in an 18% incidence of death or transplantation in the total cohort [78]. Cardiac dysfunction or ventricular arrhythmias were associated with increased mortality, which was also significantly higher in children diagnosed in the first year of life [78]. Children with normal LV diameters and normal function were at low risk for sudden death [78]. Data from the Paediatric Cardiomyopathy Registry (PCMR) suggest the specific CMP phenotype that is associated with LVNC predicts outcome in children [79]. Pediatric patients with isolated LVNC have the best outcome compared to patients with LVNC and an associated DCM/HCM [79]. 20% of cases may be familial, therefore with the diagnosis of LVNC in a child family screening is recommended [79].

A systematic review by van Waning et al. in a large cohort of genetically confirmed 561 LVNC patients found worse clinical outcome in children compared to adults [32]. The study included children with genetic syndromes, chromosomal defects, and neuromuscular symptoms. The composition of the study cohort might explain the worse clinical outcome in children caused by the severe underlying disorder [32]. Non-sarcomere non-arrhythmia CMP genes, X-linked genes, arrhythmia genes, *MYBPC3*, and *TTN* were associated with MACE in multivariate analysis [32]. The severe outcome of children with multi-systemic disorders implies that diagnostic and therapeutic procedures should be guided by age at presentation [32].

Schultze-Berndt et al. detected a higher percentage of asymptomatic children than asymptomatic adult LVNC patients in their study cohort with primary CMP [39]. Reduced LV systolic function was identified as the main risk factor for MACE in the pediatric subcohort by multivariate analysis. The LV-EF reduction in children was the main risk factor, and LV-EF had a higher independent risk than a lower BSA or increased LVEDD [39].

In summary, in pediatric LVNC lower BSA and younger age are considered risk factors for MACE [32,39]. Congestive heart failure at diagnosis and reduced LV systolic function are independent risk factors in pediatric LVNC cohorts [30,39].

### 5.2. Genetic Variants and Risk of MACE

With respect to direct genotype-phenotype correlations in LVNC, several studies produced conflicting results. In a retrospective study of LVNC detected ≤21 years of age, it was less likely to find the genetic cause in isolated LVNC than in LVNC-CMP (0% versus 12%, respectively) [80]. Isolated LVNC individuals had a negative CMP gene panel result. This genetic yield in pediatric LVNC was lower compared with previous reports in adults (41% and 29%) [28,34] and in children (38% and 28%), respectively [30,39]. This low genetic yield is difficult to explain in the context of several other studies, but might be due to the imaging criteria for the diagnosis of LVNC. The severity of LVNC by imaging criteria was not associated with a positive genetic test result in this study [80]. Anyhow, children and young adults with isolated LVNC, in the absence of a family history of CMP, were not advised to undergo CMP gene panel testing [80]. No pathogenic variants were identified in another study in adult patients with isolated LVNC in the absence of cardiac dysfunction or syndromic features [81]. A study including pediatric LVNC patients (45% of the cohort were <18 years of age) suggested that LVNC might be driven by mutational burden [50]. The number of genetic variants was associated with the extent of noncompaction and LV systolic dysfunction. Left ventricular hypertrabeculation (LVHT) and LVNC might be the result of genotype accumulation which leads to differences in the expression of the phenotype [50]. It remains elusive, if and when a mutational burden might reach a threshold for the development of LVNC and after all, impaired cardiac function and clinical symptoms.

Variants in specific genes such as in *TAFAZZIN*, *RBM20*, *Lamin A/C*, *TTN*-truncating variants and non-sarcomere genes are associated with worse outcome in LVNC [30,35,82,83]. In the study by Wang et al. pathogenic variants were identified in 38% of patients (mean age 1.8 ± 0.4 years) and were independent risk factors for MACE such as death, heart transplantation, or implantable cardioverter-defibrillator implantation [30]. Sarcomere genes accounted for 63%. *TAFAZZIN* and *MYH7* pathogenic variants were the most common. Patients with pathogenic variants showed poorer prognosis, especially those with multiple variants [30]. In the study by Schultze-Berndt et al. the genetic yield of (likely) pathogenic variants was 31%, 28% in pediatric patients, and 35% in adult patients, respectively. Mutations in *MYH7*, *TTN,* and *MYBPC3* were most prevalent in the study, very comparable to other recent reports in adult and pediatric populations [36,38]. Risk stratification for MACE, depending on the individual mutation, was not possible, neither in the pediatric nor in the adult cohort [39]. Van Waning et al. showed that in children diagnosed <1 year of age, with multiple mutations in MYBPC3, and in (probable) genetic LVNC, an increased risk of MACE was observed [38]. The risk for adverse events in sporadic (non-genetic, non-familial) children was low [38]. In summary, there is evidence that specific genetic variants have a poorer prognosis but this knowledge is not ready to be transferred to clinical decision-making.

### 5.3. Paediatric LVNC Subtypes

The impact of LVNC subtypes was systematically reviewed in a study of Van Waning et al. [32]. Adult and pediatric cases were classified as isolated LVNC, LVNC with HCM, LVNC with DCM, and LVNC with DCM and HCM. Overall, the LVNC-DCM phenotype was present in 56% and therefore the most frequent subtype. LVNC-DCM was associated with a high risk for LV systolic dysfunction and an increased risk for MACE [32]. The LVNC-HCM/DCM phenotype was detected mostly in children, was not caused by mutations in CMP genes, and lead to severe outcome [32]. In the study by Schultze-Berndt et al. a higher prevalence of LVNC-HCM in the pediatric compared to the adult subcohort was shown [39]. Patients with LVNC-HCM were younger at diagnosis, more frequently affected by CHD, and at higher risk for MACE [39].

Genetic screening to identify family members at risk for developing CMP is useful and named “cascade screening” [59]. From investigating LVNC families, van Waning et al. could predict phenotype and outcome in relatives depending on the clinical features and genotype of LVNC index cases [33]. LVNC-DCM was associated with mutations in the tail of *MYH7*, LV systolic dysfunction, increased risk for MACE, and DCM without LVNC in relatives. Mutation in the head of *MYH7,* asymptomatic or mild disease, and isolated LVNC in relatives was associated with isolated LVNC. LVNC-HCM was associated with mutation of *MYBPC3* and with HCM without LVNC in relatives [33]. These interesting data show that genetic background in families influences the cardiac phenotype associated with LVNC.

A recent study addressed the role of ion channel gene variants and correlation with arrhythmias in a large cohort of pediatric LVNC [84]. The LVNC patients with ion channel gene variants had nearly normal LV-EF and LVEDD, but showed a higher prevalence of arrhythmia phenotypes [84].

Patients with CHD and LVNC primarily present to the pediatric cardiologist and there are two possible etiologies. Pathologic remodeling induced by pressure or volume overload in CHD may lead to LVNC. Alternatively, genetic alteration may drive LVNC in CHD. In LVNC families, some relatives of affected members may have CHD or CMP with or without LVNC [26]. *MYH7* mutation was found in Ebstein anomaly associated with LVNC [6]. In LVNC with or without CHD, there was no association between the likelihood of MACE and the NC/C ratio or the number of trabeculations [85]. Van Waning et al. reported on an increased risk in children for CHD and MACE [32].

## 6. Conclusions

LVNC is a phenotype that awaits classification and can be found in a spectrum from physiologic state to mild or severe disease. Adult individuals with isolated LVNC (*LVET*) seem to have the same outcome as the healthy population. The development of better imaging technologies, specifically CMR imaging, analyzes LVNC changes more easily and might lead to better diagnosis, if used with novel imaging biomarkers. We need larger systematic population-based studies and follow-up to identify individuals at risk for developing a CMP later in life specifically in the pediatric population.

## Data Availability

Not applicable.
